# *Transversotrema hafniensis* n. sp. infection in *Poecilia reticulata* by cercariae released from *Melanoides tuberculata* in Denmark

**DOI:** 10.1186/s13028-024-00736-y

**Published:** 2024-04-02

**Authors:** Kurt Buchmann, Per Walter Kania

**Affiliations:** Department of Veterinary and Animal Sciences, Section of Parasitology and Aquatic Pathobiology, Faculty of Health and Medical Sciences, Stigbøjlen 7, DK-1870 Frederiksberg C, Denmark

**Keywords:** Disease, Ectoparasite, Fish, Ornamentals, Snail, Trematode

## Abstract

**Background:**

Exotic and ornamental fish are highly popular companion animals resulting in a significant transcontinental trade of fish, invertebrates and aquatic plants. A major issue is the diseases associated with these organisms, as they have a major impact on health of the fish in both public and private household aquaria. A secondary issue is the trade with these products, which potentially may expand the distribution area and spread a range of diseases to new habitats.

**Results:**

We here describe how *Poecilia reticulata* (guppy), produced in a private household aquarium, were invaded by cercariae of an exotic trematode released by imported *Melanoides tuberculata* snails. The fish presented with severe clinical signs (tremor, flashing, scraping of body against objects). A standard parasitological examination and morphometric identification showed scale pocket infections with a digenean trematode species within the genus *Transversotrema*. Molecular identification by PCR, sequencing and phylogenetic analyses of a 2646 bp sequence encoding ribosomal RNA (partial 18 S, ITS1, 5.8 S, ITS2, partial 28 S) was performed. The 1107 bp sequence of mitochondrial DNA (*cox1*) showed that the parasite differed from previously described *Transversotrema* species in *M. tuberculata*. Morphometrics of adult and larval specimens of this isolate also differed from previously described freshwater species within the genus. The new species was described and is named after Copenhagen, for its geographic origin.

**Conclusions:**

The genus *Transversotrema* comprises a range of species, adapted to a microhabitat in scalepockets of teleosts. A combination of morphological and molecular characterization techniques has been shown to provide a good differentiation between species. The fish were not purchased from a pet shop but produced in the home aquarium. This indicated that an infection pressure existed in the aquarium, where the source of infection was found to be exotic intermediate host snails *M. tuberculata*, which originally were imported and purchased from a pet shop. The potential spread of fish diseases associated with trade of fish and snails to new geographic regions, where climate conditions are favourable, is discussed.

**Supplementary Information:**

The online version contains supplementary material available at 10.1186/s13028-024-00736-y.

## Background

Ornamental fish are popular companion animals worldwide and the trade with fish, invertebrates and aquatic plants for use in private households has increased over the last decades from around 1 billion USD [1, 2] to several billion USD [3]. Diseases associated with these organisms may not only have a major impact on health and welfare of the fish in public and private household aquaria [4, 5], but they may also, if released into new habitats, represent a threat to endemic host species. Thus, the trade with these products may potentially expand the distribution area of non-native fish species [6], various invertebrate species [7, 8], and the parasites associated with these host organisms [5, 9–11]. Following accidental release to the surroundings, fish, snails and their diseases may be established in new habitats, where they in certain cases act as invasive species if the climate supports their survival [1, 6]. We here describe how imported non-native snails (*Melanoides tuberculata*) released cercariae of a digenean trematode within the genus *Transversotrema*, which infected locally produced ornamental fish in a Danish facility. These trematodes are characterized by their special morphology, as they are highly dorso-ventrally flattened and markedly broader than long (transversely elongate) [12]. They are ectoparasites and occupy scale pockets in a wide range of fish species [13]. This particular microhabitat may be the reason that the morphology of even remotely related different species has exhibited a limited change during evolution although species differentiation based on morphometric characters is possible [14]. The family is predominantly marine and a range of species have been reported from the Indo-Pacific region. Many, but not all, of the species can be differentiated based on their morphological characters, and the advent of molecular diagnostic techniques has been shown to improve species differentiation [14]. The transversotrematid parasite isolated from the Danish aquarium is a freshwater form with a resemblance to the species *T. patialense*, which has been reported from tropical and ornamental fish throughout the World for almost a century. The adult stage of these forms has been reported from freshwater fish in India [15–18], Sri Lanka [19], Philippines [20], Japan [21], Australia [11], California [13], and Puerto Rico [22]. The larval stage, the cercaria, released in freshwater from thiarid intermediate host snails were previously reported from India [23–25], from Thailand [26–27], from Philippines [20], from the Solomon Islands [28], Puerto Rico [22], and New Zealand [9]. Differentiation of these geographical isolates based on morphological characters alone has proven difficult, and in order to improve the species identification of the Danish isolate we have supplemented the work with a molecular identification of the parasite based on sequencing of the ribosomal RNA (partial 18 S, ITS1, 5.8 S, ITS2, partial 28 S) and mitochondrial DNA (the gene *cox1*). Based on these data we conducted a series of phylogenetic analyses. We thereby showed that the isolated trematode is a previously undescribed species within the genus *Transversotrema*, which justifies a description of the parasite as a new species. Parasites may be geographically spread with trade of parasitized ornamental fish and snails [9–11]. We therefore discuss the problems associated with transcontinental trade with parasite infected hosts in general and discuss the implications for fish health in aquacultured and natural settings.

## Methods

### Parasites and microscopical analysis

A private aquarist (Copenhagen, Denmark) observed serious clinical signs (tremor, flashing, shaking, body scratching) among her ornamental fish in her household aquarium (700 L). Two fish (guppy, *Poecilia reticulata*) were euthanized (300 mg/L MS222 Tricaine Methane Sulphonate, Sigma-Aldrich) and subjected to a standard parasitological examination using low and high magnification light microscopy (Leica MZ125, Leica DM5000B, Germany). Flatworms (Trematoda: Digenea) were found attached in scale pockets of the fish host. One fish was infected with 15 and a second with 5 digenean trematodes. The parasites released from the scale pockets exhibited waving body and ventral sucker movements (Additional file 1). The parasites were then conserved in 70% ETOH and processed for further identification. The fish were produced in the Danish household aquarium indicating that the infection had occurred locally. We based the tentative diagnosis of the digenean on its morphological appearance, as it shared characteristics of the genus *Transversotrema* [12–14, 22]. The life cycle is expected to include a mollusk (snail) as intermediate host [22–27], and the aquarist was asked to bring snails from the home aquarium facility to the diagnostic laboratory for a parasitological examination (shedding procedure of cercariae) [29]. The snails examined comprised 5 *Planorbella* (syn. *Helisoma) duryi* and 4 *Melanoides tuberculata*. They were individually incubated in 100 mL glass beakers with 10 mL freshwater for 12 h (daylight, 23 °C), whereafter the water was scrutinized for presence of cercariae under a dissection microscope (Leica MZ125, Germany). Three of the four *M. tuberculata* were found to shed cercaria. All other snails did not shed and were considered un-infected. Subsamples of cercariae were conserved in 70% ETOH for further morphometric and molecular identification. In order to investigate infectivity of the cercariae, three juvenile guppies (body length 2–3 cm, uninfected fish from own laboratory production) were placed in a 20 L infection aquarium with 9 L of aerated freshwater (temperature 23 °C, continuous aeration, green plastic plants as enrichment, natural dark-light cycle: 7 h darkness, 17 h light). Then a total of 80 cercariae were added, whereafter the infection process was observed: On day 7, 14 and 21 days post-exposure (dpe) the three guppies were examined for infection and found infected by juvenile trematodes (8, 20 and 5, respectively). The juvenile trematodes were conserved in 70% ETOH for further identification. Adult, juvenile and larval trematodes were then subjected to morphometric analysis following staining with haematoxylin and subsequent mounting in glycerine-gelatine or Aquatec (Merck, Germany). Unstained parasites were mounted in Aquatec to visualize the location of vitellaria (exhibiting yellowish colouring). Slides were examined by light microscopy (Leica DM5000B, Germany), morphological characteristics recorded, micro-photos recovered (Leica MC170 HD, Leica Microsystems, Singapore) and a line drawing prepared.

### Molecular analysis

#### Isolation of genomic DNA, PCR, and sequencing

Ethanol-conserved parasites (adult parasites from fish in the household aquarium, cercariae released from the snail, juvenile parasites from experimentally infected fish) were subjected to DNA-purification and a diagnostic PCR performed using primers (Table 1), amplifying the sequences encoding ribosomal RNA (18 S, ITS1, 5.8 S, ITS2, 28 S) [30–32] and mitochondrial DNA (*cox1*) [33–34]. Genomic DNA was purified by DNA using QIAamp DNA Mini Kit (cat.no.51,306, Qiagen, Denmark). PCR was performed using a BIORAD T100 thermal cycler (BIORAD, Denmark) in a 60 µl reaction (3 u of BIOTAQ DNA Polymerase (cat.no. BIO-21,060, Nordic BioSite, Denmark), 10 mM dNTP (cat no. 4,311,235, Thermo Fisher Scientific, Denmark). PCR conditions: predenaturation at 95 °C for 5 min, and for rRNA/ITS this first step was followed by 40 cycles of denaturation at 95 °C for 30 s, annealing at primer specific temperature for 30 s and elongation at 72 °C for a primer specific time, and finally a post elongation step at 72 °C for 5 min (Table 1). With regard to the PCR of mt DNA, the gene *cox1*, a touch-down procedure was applied: annealing at 53 °C 2 cycles, 51 °C 2 cycles, 50 °C 2 cycles, 49 °C 3 cycles, 48 °C 3 cycles, 47 °C 3 cycles, 46 °C 35 cycles (Table 1). The products were visualized by ethidium bromide following 1.5% agarose gel electrophoresis (cat.no. 10,264,544, Thermo Fisher Scientific, Denmark). They were then purified using illustra™ GFX™ PCR DNA and Gel Band Purification Kit (cat.no. 28-9034-71, VWR International, Denmark) and sequenced at Macrogen (Netherlands). The obtained sequences were subjected to analysis using CLC Main Workbench v20.0.4 (QIAGEN Aarhus A/S, Denmark) and GenBank using BLAST.

**Table 1:** Primers and PCR conditions. Primers for the rRNA and ITS regions [30–32] and for *cox1* [33–34] were applied. Pre-denaturation at 95 °C for 5 min, followed by 40 cycles of denaturation at 95 °C for 30 s, annealing at primer specific temperature for 30 s and elongation at 72 °C for a primer specific time, and finally a post elongation at 72 °C for 5 min. For the mtDNA PCR a touchdown procedure was applied: annealing at 53 °C 2 cycles, 51 °C 2 cycles, 50 °C 2 cycles, 49 °C 3 cycles, 48 °C 3 cycles, 47 °C 3 cycles, 46 °C 35 cycles

Product	Primer	Sequence	Specific PCR condition
rRNA/ITS, 5’ end	BD1	gaggaattcctggtaagtgcaag	Annealing at 58 °C Elongation for 150 s.
	NC2	ttagtttcttttcctccgct	
rRNA/ITS, 3’ end	NC5	gtaggtgaacctgcggaaggatcatt	Annealing at 55 °C Elongation for 75 s.
	NLR1270	ttcatcccgcatcgccagttc	
*cox1*	MplatCOX1dF	ttwcnttrgatcataag	Annealing, Touchdown Elongation for 120 s.
	CO1-R trema	caacaaatcatgatgcaaaagg	

### Phylogenetic analysis

The software package CLC Main Workbench was used for the phylogenetic analysis. The general workflow was alignment using Clustall W [35], testing of model using four different statistical analyses (Hierarchical likelihood ratio test (hLRT), Bayesian information criterion (BIC), Minimum theoretical information criterion (AIC), Minimum corrected theoretical information criterion (AICc)), and generating phylogenetic trees by Maximum Likelihood Phylogeny. In all cases, the GTR + G + T were proposed as the best nucleotide substitution model. Maximum Likelihood Phylogeny was performed with initial trees generated using the UPGMA construction method (parameters: Transition / transversion ratio = 2.0, Include rate variation = Yes, Number of substitution rate categories = 4, Gamma distribution parameter = 1,0, Estimate substitution rate parameter(s) = Yes, Estimate topology = Yes, Estimate gamma distribution parameter = Yes, 100 bootstraps). Then Neighbor Joining trees were constructed using the UPGMA trees as initial trees and using the same parameters except that 1000 bootstraps were performed [36]. Table 2 indicates the sequences used for the analyses. All sequences were included during the alignment, model testing, and construction of trees. The aim of these analyses was to establish the relationship of the new species to other Transversotrematidae species (Table 2) and not to investigate further details of the genus. With regard to IT2, the alignment was first conducted with the relatively well conserved 3’ end of 5.8 S rRNA and 5’ end of 28 S rRNA to ensure proper alignment of the ITS2 borders. Thereafter the 5.8 S rRNA and 28 S rRNA parts were removed (whereby model testing and phylogram construction include ITS2 only).

**Table 2:** Sequences used in phylogenetic analyses. Sequences were adopted from [14]. Redundant sequences were excluded and sequences of *T. hafniensis* n. sp., *T. patialense*, *P. steeri*, and *A. longa* were included (indicated by GenBank accession numbers). The latter served as an outgroup.

Species	cox1	ITS2	28 S
^a^ Number of sequences from [14]	236 sequences	41 sequences	89 sequences
^b^ Number of species from [14]	35 species	35 species	35 species
*Transversotrema hafniensis* n. sp.	OR432511	OR436781	OR436781
*Transversotrema patialense*	OP066719	OP08873	OP099865
*Prototransversotrema steeri*	NA	GU174458	AY222184
*Azygia longa*	KM538080	KT808319	KC985234

NA indicates not available. ^b^ indicates number of species represented in ^a^

## Results

### Morphometric analysis and description

**Fig. 1:**
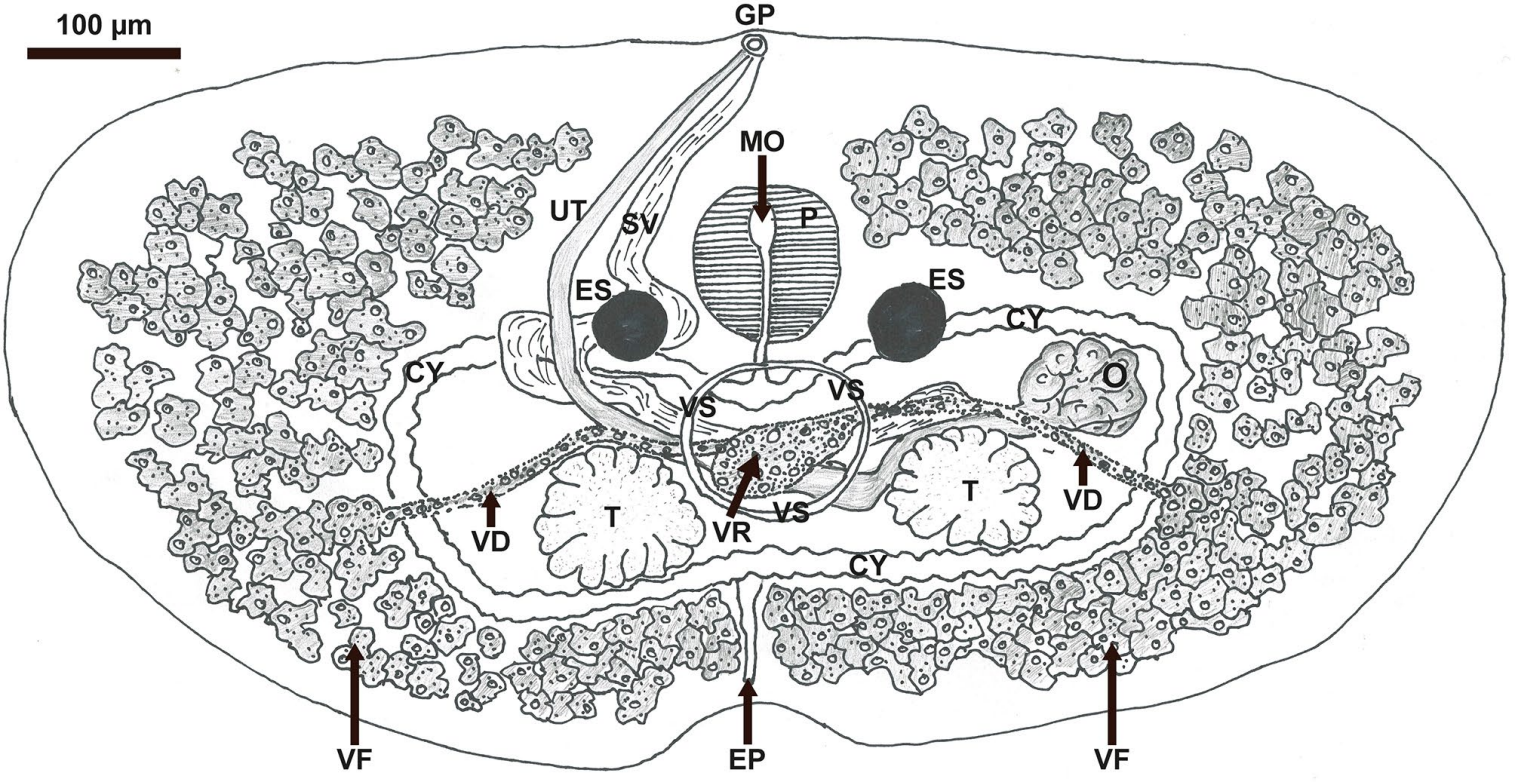
Line drawing of *Transversotrema hafniensis* n. sp. ventral view. GP: common gonopore, SV: seminal vesicle, UT: uterus, M: mouth, P: pharynx, ES: eye spot, VS: ventral sucker, CY: cyclocoel, O: ovary, T: testis, EP: excretory pore, VF: vitelline follicles, VR: vitelline reservoir, VD: vitelline duct

**Fig. 2:**
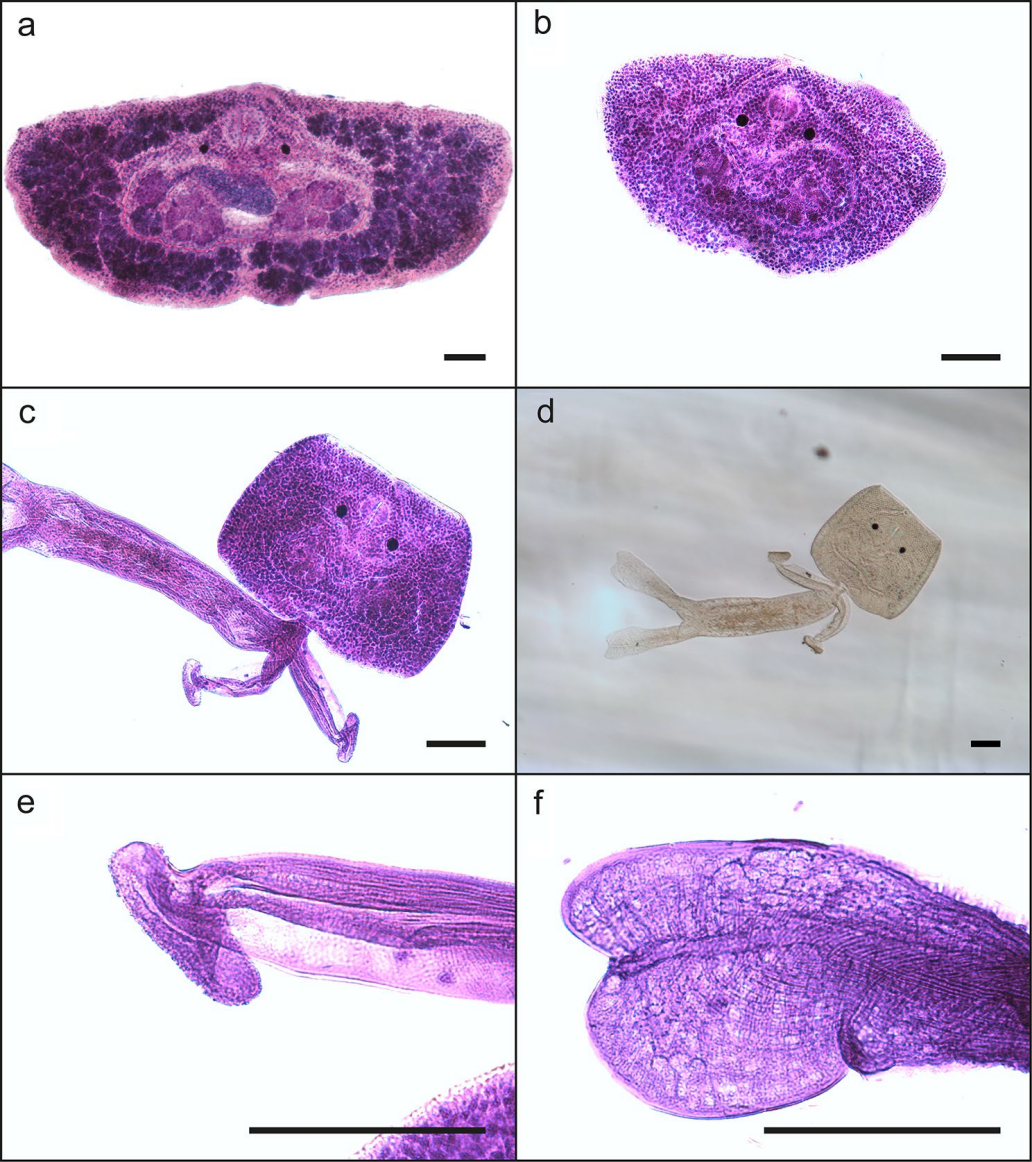
LM micrographs of a *Transversotrema hafniensis* n. sp. mounted in glycerine-gelatine. All specimens stained by haematoxylin except 2d (unstained). Scale bar 100 μm. 2**a**. Adult trematode, 2**b**. Cercarial body detached from the cercarial tail. 2**c**. Cercaria from *M. tuberculata* showing body proper and anterior armlike appendages. 2**d**. Cercaria *in toto*. 2**e**. Anterior cercarial tail appendage. 2**f**. Distal cercarial tail appenage

The morphology of five adult parasites (from the fish) and four larval stages (cercariae from the snail) documented that the parasite was a digenean trematode within the genus *Transversotrema*. These adult parasites are dorsoventrally flattened but are crescent-shaped, as the length is significantly shorter than the width (transversely elongate). The analyses performed indicated that the isolate represented a new species. The morphology is shown in Figs. 1 and 2. Morphometrics of the adult body, compared to other freshwater species within the genus *Transversotrema*, are shown in Table 3. Characteristics of the cercaria, compared to other freshwater species within the genus *Transversotrema*, are shown in Table 4.

**Table 3:** Morphometric characters of adult *Transversotrema hafniensis* n. sp. specimens. Comparison with other freshwater species within the genus *Transversotrema* based on measurements (range (mean) in microns (µm). Figures in bold show characteristic size differences of *T. hafniensis* n. sp. compared to other freshwater species within the genus

Species name or synonym	Transversotrema hafniensis n. sp.	T. patialense	T. laruei	T. patialense	T. soparkari	T. chackai	T. chauhani	T. patialense	T. patialense	T. patialense	T. patialense
Geographic location	Denmark	Sri Lanka	Philippines	India	India	India	India	Australia	Japan	California	Puerto Rico
Author	Present study	Crusz & Sathananthan (1960)	Velasquez (1961)	Murty & Hanumantha (1968)	Pande & Shukla (1972)	Mohandas (1973)	Agrawal & Singh (1981)	Cribb et al. (1992)	Maneepitaksanti & Nagasawa 2012	Womble et al. 2015	Perales Macedo et al. 2022
Reference	–	19	20	15	16	17	18	12	21	13	22
Body length	565–620 (589)	306–323	250–550 (390)	312–375 (352)	375–675	400–460	300–440	361–503 (420)	300–405 (339)	400–530 (461)	224–613 (417)
Body width	**1100–1380 (1240)**	612–700	460–900 (680)	680–732 (720)	735–1437	626–950	640–710	789–947 (882)	640–870 (730)	820–1060 (967)	567–1212 (758)
Pharynx Length	**100–110 (104)**	48–50	40–50 (45)	36–52	58–97	55–70	40–50	59–79 (68)	50–68 (58)	63–88 (72)	34–96 (63)
Pharynx width	**90–100 (98)**	–	–	36–60	58–115	–	50–60	59–69 (64)	50–70 (56)	65–90 (77)	37–104 (68)
Ventral sucker Length	100–120 (110)	102	70–90 (80)	60–84	119–141	100–135	28–30	119–151 (140)	90–120 (110)	108–128 (117)	60–120 (96)
Ventral sucker width	100–120 (110)	–	–	–	–	–	–	131–156 (147)	97–118 (108)	115–133 (121)	63–125 (99)
Egg length	120–170 (145)	–	125–150 (138)	112–130 (120)	140–178	125–160	30–33	119–141 (129)	120–125 (123)	110–143 (131)	107—189 (141)
Egg width	80–110 (95)	–	46–81 (63)	44–60 (56)	68–109	50–75	25–29	67–75 (73)	90–107 (99)	38–48 (44)	41–96 (70)

**Table 4:** Morphometric characters of *Transversotrema hafniensis* n. sp. cercariae. Comparison with other freshwater species within the genus *Transversotrema* and at present considered *T. patialense.* Synonym in parenthesis. Measurements shown as range (mean) in microns (µm)

Species name and synonym	Transversotrema hafniensis n. sp.	T. patialense	T. patialense	T. patialense (T. laruei)	T. patialense	T. patialense	T. patialense	T. patialense	T. patialense
Geographic location	Denmark	India	Solomon Islands	Philippines	India	India	Thailand	Thailand	Puerto Rico
Author	Present study	Soparkar (1924)	Olivier (1947)	Velasquez (1961)	Rao and Ganapati (1967)	Nadakal et al. (1969)	Ukong et al. (2007)	Krailas et al. (2014)	Perales Macedo et al. 2022
Reference	–	23	28	20	25	24	26	27	22
Body length	300–330 (312)	374–459	240–310 (270)	280–430 (350)	430–470	440–590 (505)	200–480 (301)	280–510 (370)	295–501 (392)
Body width	480–560 (507)	697–782	260–490 (507)	480–700 (590)	570–600	720–1020 (835)	338–750 (508)	425–670 (574)	399–824 (626)
Pharynx Length	35–65 (48)	58–78	45–55	50	42	50–80 (60)	30–55 (49)	25–57	24–67 (54)
Pharynx width	30–60 (45)	–	–	–	57	–	10–63 (46)	28–51 (37)	26–75 (59)
Genital pore length	13–25 (19)	–	–	–	–	–	10–18 (14)	12–18 (15)	12–28 (21)
Genital pore width	10–30 (20)	–	–	–	–	–	–	12–18 (15)	16–34 (25)
Tail stem length	420–559 (462)	580–595	400–430 (420)	400–430 (420)	494	500–630 (567)	225–600 (351)	254–570 (360)	353–640 (490)
Tail-stem width	130–145 (138)	102	90–120 (110)	90–120 (110)	114	110–160 (136)	35–190 (81)	50–185 (98)	54–133 (97)
Furca length	250–280 (265)	340–374	180–250 (220)	180–250 (220)	–	300–360 (313)	125–300 (184)	145–310 (204)	173–338 (271)
Furca width	95–120 (106)	135	70–90 (80)	70–90 (80)	–	90–110 (103)	20–100 (50)	45–110 (66)	37–127 (80)
Appendage length	200–220 (210)	110–130	120–180 (150)	120–180 (150)	–	240–280	113–280 (154)	120–250 (160)	167–299 (207)
Appendage width	40–50 (45)	–	30–50 (40)	30–50 (40)	–	60–90 (76)	15–70 (39)	18–72 (47)	28–77 (54)

*Transversotrema hafniensis* n. sp.

Type host: Guppy, *Poecilia reticulata*.

Type-locality: Freshwater aquarium, Copenhagen, Denmark.

Type material: Holotype and paratypes were deposited in the Natural History Museum of Denmark. Holotype: Cat. No. NHMD-1,651,248. Paratypes (adult parasites): Cat. Nos. NHMD-1651249-16511251. Paratypes (cercariae): Cat. No. NHMD-1,651,252.

Site in host: Beneath scales (scale pockets).

GenBank accession data: Sequences obtained have been deposited in GenBank: For the rRNA/ITS region (GenBank acc.no. OR436781) and for the mitochondrial cytochrome c oxidase subunit I (*cox1*) gene (GenBank acc.no. OR432511).

Etymology: The species name refers to the location of isolation Copenhagen, Denmark, which is Hafnia in latin.

Description of adult (Figs. 1 and 2), based on 5 whole mounts of adult specimens:

Body transversely elongated. Tegumental spines prominent. Eyespots prominent, paired, central in anterior part of the body, level with lower edge of pharynx, no pigment evident other than in eyespots. Ventral sucker posterior to eyespots. Mouth mid-ventral at upper half of pharynx. Pharynx prominent. Oesophagus directed posteriorly from pharynx towards cyclocoel. Intestinal bifurcation located dorsal to upper part of ventral sucker. Cyclocoel enclosing two testes, deeply lobed, ovary sinistral to left testis. Common genital pore in midline of anterior margin of worm. Vitelline follicles densely assembled and distributed anterior, posterior and lateral to cyclocoel, vitelline ducts passing in midline of the body extending from lateral groups of vitelline follicles, merging to vitelline reservoir in midline of body. Eggs tanned, unembryonated with no operculum. Excretory vesicle opens posteriorly at small notch in middle of posterior margin of body.

Description of cercaria (Fig. 2), based on four specimens:

*Transversotrema hafniensis* n. sp.

Host: *Melanoides tuberculata*.

Locality: Freshwater aquarium, Copenhagen, Denmark.

Material: Paratypes (cercariae) were deposited in the Natural History Museum of Denmark: Cat. No. 16,511,252.

GenBank accession data: Sequences obtained have been deposited in GenBank: For the rRNA/ITS region GenBank acc.no. OR436781) and for the mitochondrial cytochrome c oxidase subunit I (*cox1*) gene (GenBank acc.no. OR432511).

Cercarial body transversely elongated. Two eyespots prominent, located in upper part of the body midline. Pharynx prominent. Cyclostome conspicuous. Common genital pore in midline of anterior margin of body. Vitelline follicles not present. Gonad anlagen present. Cercarial tail trunk equipped with two arm-like appendages, distally placed spine-equipped swimming pads (Fig. 2e) (Fig. 2f).

### Molecular diagnosis (subheading 5)

In the case of the rRNA/ITS region, a 2646 bp long sequence (GenBank acc.no. OR436781) was obtained by combining the result of sequencing two PCR product having a 1011 bp long overlap. The sequence was analysed using the internet source Rfam [36] and consisted of the 3’ end of the ribosomal RNA 18 S (233 bp), ITS1 (520 bp), 5.8 S (153 bp), ITS2 (243 bp) and finally the 5’ end of 28 S (1497 bp). BLAST at GenBank optimized for “Somewhat similar sequences” showed highest identity toward Puerto Rico isolate of *T. patialense*, GenBank acc.no. OP088732 (5.8 srRNA: 98.5%, ITS2: 84.5% and 28 S rRNA: 88.1%). In the case of the mitochondrial cytochrome c oxidase subunit I (*cox1*) gene, a 1090 bp sequence (GenBank acc.no. OR432511) was obtained. BLAST at GenBank optimized for “Somewhat similar sequences” showed highest identity (83.5%) toward the Puerto Rico isolate of *T. patialense* (GenBank acc.no OP066719). In general, the results of the phylogenetic analysis are presented as phylograms based on Maximum Likelihood analysis using initial trees generated by the UPGMA method (100 bootstraps) and final trees generated by the Neighbor Joining method (1000 bootstraps).

**Fig. 3:**
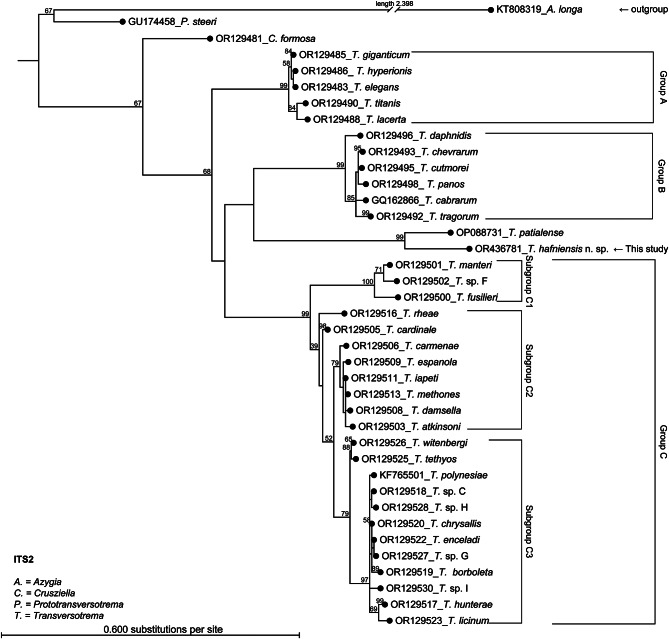
Phylogenetic tree of ITS2 sequences. The tree was based on Maximum Likelihood Phylogeny using the Neighbor Joining method (1000 bootstraps) and all specimens. The tree was constructed using all specimens. However, each leaf node represents one species, but it may represent several specimens. Only bootstrap values > 50% are presented. The radial tree is provided as Additional file A2

**Fig. 4:**
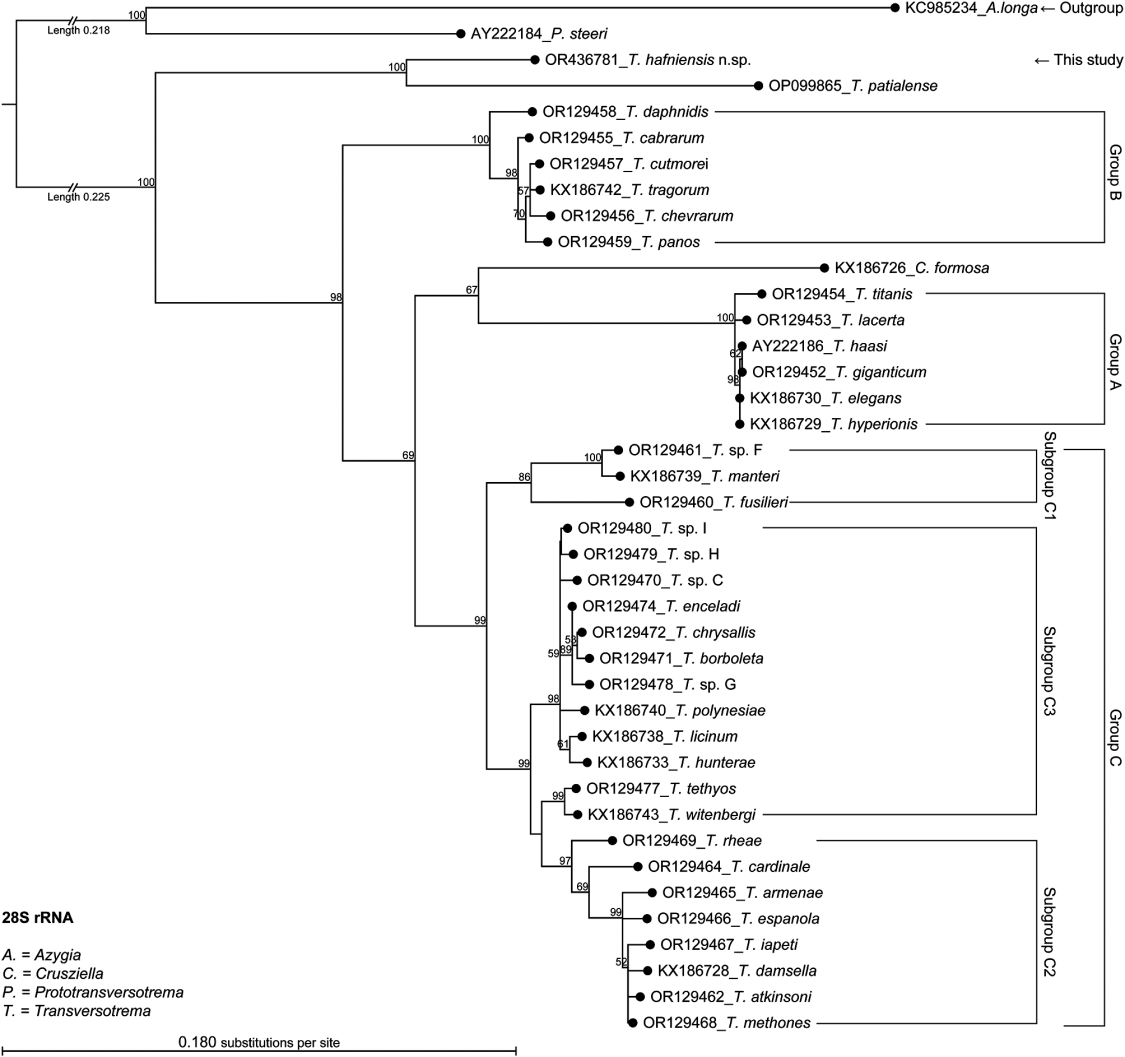
Phylogenetic tree of 28 S rRNA sequences. The tree was based on Maximum Likelihood Phylogeny using the Neighbor Joining method (1000 bootstraps) and all specimens. However, each leaf node represents one species, but it may represent several specimens. Only bootstrap values > 50% are presented. The radial tree is provided as Additional file A3

**Fig. 5:**
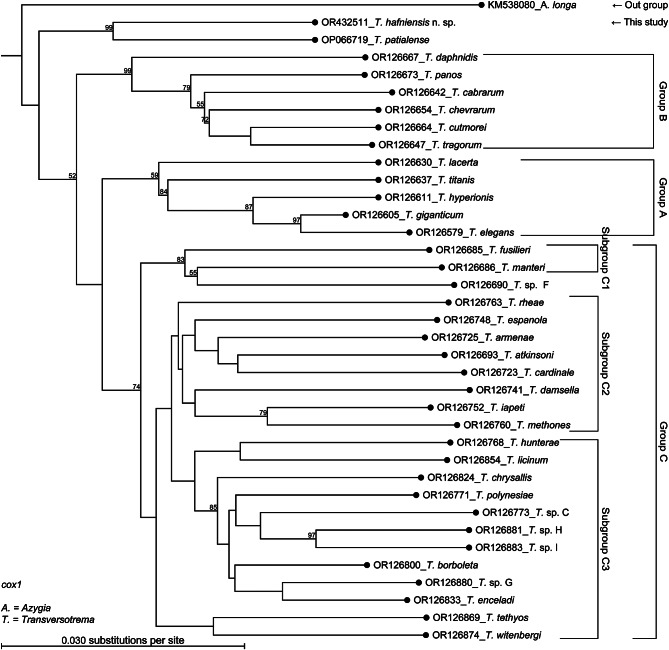
Phylogenetic tree of the mitochondrial *cox1* sequences. The tree was based on Maximum Likelihood Phylogeny using the Neighbor Joining method (1000 bootstraps) and all specimens. However, each leaf node represents one species, but it may represent several specimens. Only bootstrap values > 50% are presented. The radial tree is provided as Additional file A4

The phylograms are presented as phylogenetic trees as Fig. 3 (ITS2), Fig. 4 (28 S rRNA), and Fig. 5 (*cox1*) and as radial phylograms as additional files A2, A3, and A4. The alignments are available in the fasta format (.fasta) as additional files A5, A6, and A7. The main difference between the phylogenetic analysis of this study and that of a recent study [14] was, that these authors did not use an outgroup in the case of *cox1* and used *Crusziella formosa* as an outgroup in the cases of ITS2 and 28 S rRNA, whereas the present study included the evolutionary more distant *Azygia longa* in all three cases. This study also included sequences of *Transversotrema patialense* (ITS-region, 28 S, and *cox1*), sequences of *Prototransversotrema steeri* (ITS2 and 28 S rRNA only, as *cox1* sequences were not available), and a sequence of *Transversotrema haasi* (28 S). The grouping of *Transversotrema* species was adopted from [14]. A general feature of all obtained phylogenetic trees (Figs. 3 and 4, and 5) was the relative position of *T. hafniensis* n. sp., which clustered strongly together with *T. patialense* (bootstrap value ≥ 99) and with higher affinity towards Group B species than towards Group A and Group C species. Seen overall, *T. hafniensis* and *T. patialense* clustered together clearly separated from the outgroups and other Transversotrematidae species including *P. steeri*. As indicated by the BLAST results mentioned above, sequences of *T. hafniensis* n. sp. were most similar to those of *T. patialense*, but similarities were nonetheless relatively low. The bootstraps values at nodes joining *T. hafniensis* n. sp. and *T. patialense* were high.

#### Differential diagnosis

Adult morphology. *T. hafniensis* n. sp. differs from other hitherto described freshwater species within the genus *Transversotrema* with regard to the larger size of body and pharynx (length 100–110, width 90–100 μm), compared *T. patialense* (California isolate) (mean length 72 and width 77 μm) and the Puerto Rico isolate (mean length 63 and width 68 μm). Measurements of other isolates showed even lower values (Table 3).

Cercarial morphology. *T. hafniensis* n. sp. cercaria differs from other previously described freshwater species. Body length of *T. hafniensis* n. sp. (mean 312 μm) and width (mean 507 μm) is shorter than the Puerto Rico *T. patialense* isolate (392 μm and 626 μm, respectively). The tail width is also larger (138 μm) compared to the Puerto Rico isolate (97 μm) (Table 4).

*T. hafniensis* n. sp. differs from the type species of *T. patialense* [23] by having shorter tail stem length (420–559 μm compared to 580–595 μm) and furca length (250–280 μm compared to 340–374 μm) (Table 4). Molecular differences to *T. patialense* are shown in Table 5.

**Table 5:** Comparison of molecular characteristics of *T. hafniensis* n. sp. and *T. patialense*. Based on Clustal W alignments using the two species in question. All numbers are nucleotides in bp (base pairs).

Gene	Species	GenBank acc.no	^a^ Length	Gaps	^b^ Differences	Identities
ITS2	*T. hafniensis* n. sp.	OR436781	415	0	67	312
	*T. patialense*	OP088731	379	36		
28 S	*T. hafniensis* n. sp.	OR436781	1195	1	136	1059
	*T. patialense*	OP099865	1195	1		
*cox1*	*T. hafniensis* n. sp.	OR432511	474	0	86	388
	*T. patialense*	OP066719	474	0		

^a^ Length is without gaps. ^b^ Differences includes gaps

### Clinical signs and infection (subheading 6)

The aquarium holder, having a local production of guppies, observed severe clinical signs among a stock of guppies, which had been born and subsequently kept in the aquarium for two months. The fish presented with tremor, flashing and were vigorously shaking the body and scratching the body surface against objects (plants, ornamentation, bottom gravel) in the aquarium. When brought to the fish pathology laboratory the parasitological examination of the fish showed an infection with transversotrematids (see parasitological results above).

The examination of snails, sampled in the same production aquarium, were subjected to shedding procedures to reveal active infection. This documented a steady release of infective cercariae, which indicated existence of an infection pressure in the production facility. This potential was confirmed by conducting successful experimental infections of guppies in the laboratory. One month after the tentative diagnosis the client initiated bath treatment of the infected 700 L home aquarium (temperature 25 °C) using praziquantel 10 mg/L, and already within two days post drug administration (dpd) the clinical signs disappeared from the fish. They became calm, swam quietly and did not present with tremor or flashing. A total of 11 guppy fish (body length 3–4 cm) from the treated home aquarium was then sampled and brought to the laboratory for examination at 14 and 60 dpd, respectively. All 11 fish were negative for *Transversotrema*. This indicates that the praziquantel treatment had been effective against *Transversotrema* on fish. No young stages were found neither, indicating absence of reinfection, and thereby that also the infection of snails, or at least release of infective cercariae, in the aquarium had been affected by praziquantel administration. Six months post-treatment (180 dpd) the aquarium holder reported that clinical signs had not re-appeared.

## Discussion

The present work describes the isolation of a pathogenic trematode from scale pockets of ornamental fish (*P. reticulata*) in a Danish aquarium. The fish in the facility were infected by cohabitation with infected *M. tuberculata* snails releasing infective cercariae. Subsequent infection studies using snails from the production aquaria confirmed that the parasites in fish and snails were identical. This was documented by sequencing DNA encoding rRNA/ITS and mtDNA sequences from the different stages (cercaria, juvenile, adult). Morphometric analyses of the parasite indicated that the species belong to the family Transversotrematidae and the genus *Transversotrema* [12], but due to a high degree of morphological similarity between species we supplemented the work with molecular analyses [14]. The phylogenetic analyses using ITS1, 28 S rRNA, and *cox1* mtDNA supported the view that the isolated parasite was a new species closely related to *T. patialense*, the only freshwater species in the genus, and more distantly related to other Transversotrematidae, including members of the freshwater genus *Prototransversotrema*. When comparing the morphological characteristics of adult parasites of the Danish isolate with previously published studies from India [15–18], Sri Lanka [19], Philippines [20], Japan [21], California [13], and Puerto Rico [22] it was noted that the dimensions of the Danish isolate were larger with regard to body size (width) and pharynx (length and width). The cercariae of the Danish isolate differed from the original description by Soparkar [23] by having a shorter body, shorter and wide tail stem, shorter and narrower furcae and longer caudal appendages. The Danish isolate differed also from the Puerto Rico isolate of *T. patialense* [22] by exhibiting shorter body length and width but larger tail width. The Danish isolate was then named *T. hafniensis* n. sp. signifying the geographic location (Hafnia, latin for the city Copenhagen in Denmark). We have visualized the phylogeny by trees (in the main document) and as radial phylograms (additional files). We thereby established the phylogenetic relationship of *T. hafniensis* n. sp. but did not elaborate further on the *Transversotrema* taxon with respect to geographical origin, as this task already was addressed in a recent comprehensive study [14]. The phylogenetic analyses strongly supported the identification of *T. hafniensis* n. sp. as a new species closely related to *T. patialense*, and closest to Group B. Clear affiliation of these two species to any of the groups defined earlier [14, 37] was not found. Future phylogenetic analyses could benefit from including ITS1, either separately or as a part of a longer product, i.e. combined ITS1 -5.8 S rRNA - ITS2–28 S rRNA, which may be produced by PCR using the primers D1 and NLR1270 (Table 1). The results indicated that *T. hafniensis* n. sp. and *T. patialense* may form an independent clade in the family Transversotrematidae. With respect to *cox1*, the variations within groups, relative to the variation between the groups, was greater than for ITS2 and 28 S rRNA. Thus, the discrimination between the species was more evident and the phylogenetic tree of *cox1* was the most useful one. Therefore, future studies should investigate the *cox1* sequence of *P. steeri*, which is not available at present. This could further elucidate the indication seen in the phylograms of ITS2 and 28 S of *P. steeri* being a common ancestor of the family Transversotrematidae. The present study has shown that the intermediate host snail *M. tuberculata* can carry two species, *T. hafniensis* n. sp. and *T. patialense.* Formerly only *T. patialense* was known from this snail, because other freshwater species were considered synonyms [12], but the advent of molecular tools have made it possible to obtain a better resolution. However, even on the morphometric level the cercariae of Danish isolate differ from the original description of *T. patialense* [14] and supports the existence of a new species. The isolate from Puerto Rico [22] also differed from the type material but to a lesser degree, which could be interpreted as this being conspecific with, or at least closely related to the type species. The study suggests that future use of combined morphometrics and molecular methods may be able to differentiate further freshwater species within the genus in line with the impressive expansion seen for marine species [14].

The fish examined in the present study were produced in the local home aquarium, and the source of infection was imported snails (carrying parasites) purchased from a pet shop. In a fish health context this should be emphasized, because the parasites elicited clinical signs in the fish host. The recorded clinical impact of the infection has not previously been described, despite the use of this host/parasite model in several biological studies on life cycle details [38–41]. Mild pathological reactions in ornamental fish elicited by *T. patialense* were mentioned previously [38], but the marked reactions, similar to the ones induced by *T. hafniensis* n. sp., were not presented before. The reason for lack of previous pathological reports is unknown, but it cannot be excluded that *T. hafniensis* n. sp. has higher virulence than previously studied species. Future controlled infection studies, including various geographical isolates and employing histopathological, immunohistochemical and molecular (gene expression) techniques, may elucidate the pathogenesis of this type of parasites.

Trade with infected fish may spread diseases, including viral, bacterial and parasitic pathogens (protozoans, metazoans). Trematodes in ornamental fish, such as *Centrocestus formosanus*, may play a role as well [4], although the complex three host life cycle reduces the risk of further spread. In general the issue with spread of diseases is not likely to decrease, as the trade is considerable (5.88 billion USD in 2022) and is expected to increase even further at an annual growth rate of 8.5% until 2030 [3]. Increased focus on better examination of imported fish and snails, could prevent introduction of infected material before release on local markets [5–7]. The species *T. patialense* sensu lato (with a two host life cycle) has a broad host range and is able to colonize several fish species [40]. The risk associated with this group of parasites is minor compared to other pathogens with direct life cycles (protozoans, monogeneans), but it cannot be excluded that spread of snails releasing *Transversotrema* cercariae may represent a risk for endemic fish species [11]. This has not yet been documented, but ornamental fish have been shown released into European waters [6] and the intermediate host *M. tuberculata* has already been established in localized areas (central Europe and the Netherlands), in which heat energy release from geothermic sources or industrial activity occurs [42].

## Conclusions

A new species of an ectoparasitic digenean trematode within the genus *Transversotrema* was isolated from ornamental fish in Copenhagen, Denmark, and it was described as *T. hafniensis* n. sp. specifying its origin. It was isolated from ornamental fish (*P. reticulata*), exhibiting clinical signs, which disappeared following praziquantel bath treatment. It was shown that intermediate host snails *M. tuberculata* in the aquarium released infective cercariae causing infection of naïve fish. The description was based on morphometric and molecular analyses of sequences encoding rRNA, ITS and mtDNA. We discuss the risk associated with accidental release of infected fish and intermediate host snails in local habitats where local climatic conditions may support survival of hosts and parasites.

### Electronic supplementary material

Below is the link to the electronic supplementary material.

Supplementary Material 1

Supplementary Material 2

Supplementary Material 3

Supplementary Material 4

Supplementary Material 5

Supplementary Material 6

Supplementary Material 7

## Data Availability

The datasets used and/or analysed during the current study are available from the corresponding author on reasonable request. Holotype and paratypes were deposited in the Natural History Museum of Denmark. Holotype: Cat. No. NHMD-1651248. Paratypes (adult parasites): Cat. Nos. NHMD-1651249-16511251. Paratypes (cercariae): Cat. No. NHMD-1651252. Sequences obtained have been deposited in GenBank: For the rRNA/ITS region GenBank acc.no. OR436781) and for the mitochondrial cytochrome c oxidase subunit I (cox1) gene (GenBank acc.no. OR432511).

## Declarations

### Prior publication

Data have not been published previously.
