# Supplementary figures and images for: Transversotrema hafniensis n. sp. infection in Poecilia reticulata by cercariae released from Melanoides tuberculata in Denmark
Source: Acta Vet Scand. 2024 Apr 2;66:15. doi: 10.1186/s13028-024-00736-y (PMC10988963; doi:10.1186/s13028-024-00736-y)

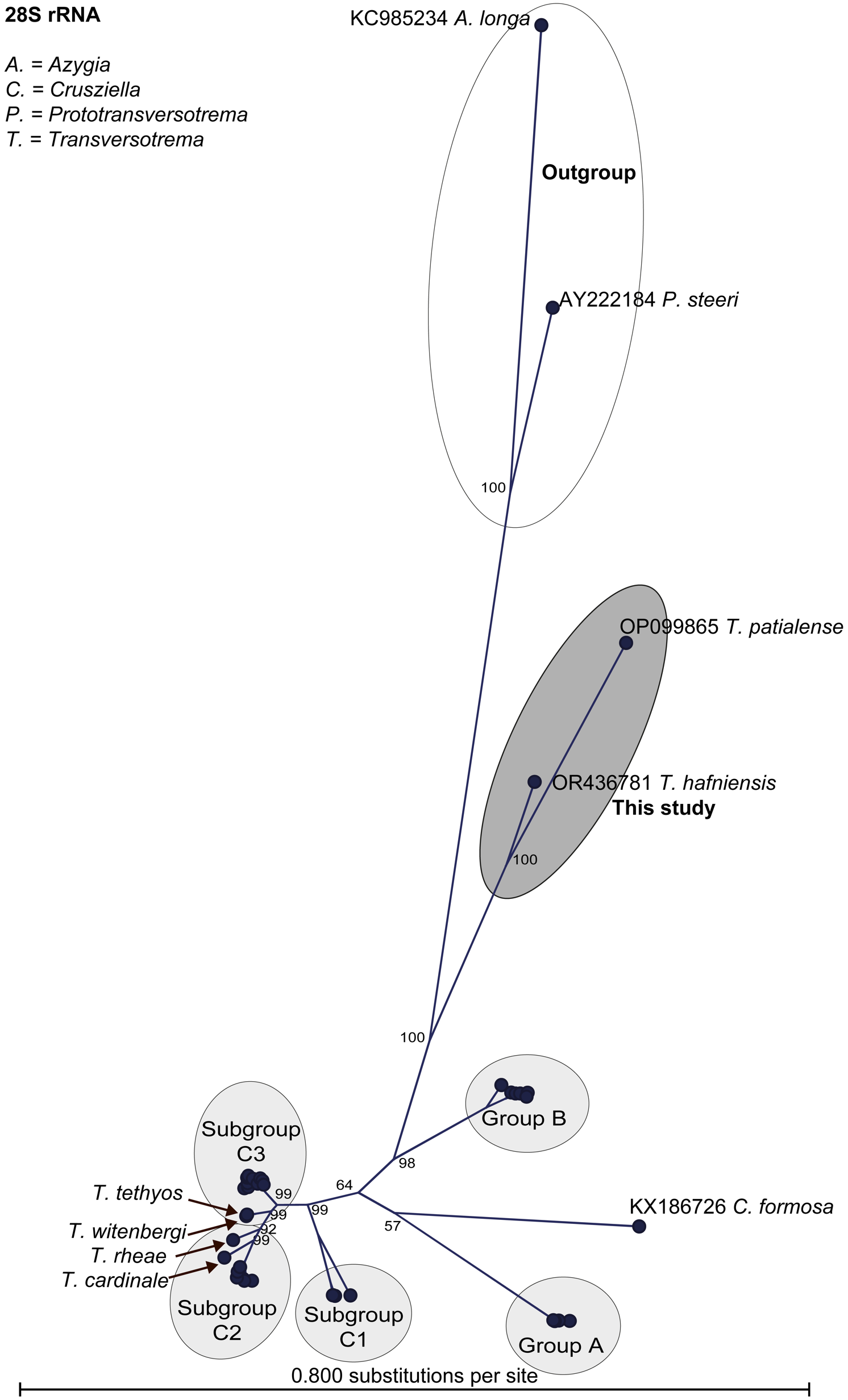

Supplement: Supplementary file 3 — Supplementary Material 3 [file 13028_2024_736_MOESM3_ESM.tif]
